# Pathology of Puumala Hantavirus Infection in Macaques

**DOI:** 10.1371/journal.pone.0003035

**Published:** 2008-08-21

**Authors:** Tarja Sironen, Jonas Klingström, Antti Vaheri, Leif C. Andersson, Åke Lundkvist, Alexander Plyusnin

**Affiliations:** 1 Department of Virology, Haartman Institute, University of Helsinki, Helsinki, Finland; 2 Swedish Institute for Infectious Disease Control, and MTC, Karolinska Institutet, Stockholm, Sweden; 3 HUSLAB and Department of Pathology, Haartman Institute, University of Helsinki, Helsinki, Finland; University of California San Francisco, United States of America

## Abstract

Hantaviruses are globally important human pathogens that cause hemorrhagic fever with renal syndrome and hantavirus pulmonary syndrome. Capillary leakage is central to hantaviral diseases, but how it develops, has remained unknown. It has been hypothesized that the pathogenesis of hantavirus infection would be a complex interplay between direct viral effects and immunopathological mechanisms. Both of these were studied in the so far best model of mild hemorrhagic fever with renal syndrome, i.e. cynomolgus macaques infected with wild-type Puumala hantavirus. Viral RNA detected by *in situ* hybridization and nucleocapsid protein detected by immunohistochemical staining were observed in kidney, spleen and liver tissues. Inflammatory cell infiltrations and tubular damage were found in the kidneys, and these infiltrations contained mainly CD8-type T-cells. Importantly, these results are consistent with those obtained from patients with hantaviral disease, thus showing that the macaque model of hantavirus infection mimics human infection also on the tissue level. Furthermore, both the markers of viral replication and the T-cells appeared to co-localize in the kidneys to the sites of tissue damage, suggesting that these two together might be responsible for the pathogenesis of hantavirus infection.

## Introduction

Hantaviruses (genus *Hantavirus*, family *Bunyaviridae*) are rodent-borne viruses that have a tripartite genome of negative-strand RNA [1]. They are the causative agents of hemorrhagic fever with renal syndrome (HFRS) in Eurasia, and hantavirus pulmonary syndrome (HPS) in the Americas. A mild form of HFRS called nephropathia epidemica (NE) is caused by Puumala virus (PUUV) [2]. Although capillary leakage is the hallmark of hantaviral diseases, its detailed mechanisms remain largely unknown. Hantaviruses commonly infect endothelial cells [3], which is thought to play a key role in the development of HFRS and HPS. Increased levels of serum perforin, granzyme B, and caspase-cleaved cytokeratin-18 have been reported in HFRS patients, suggesting some tissue damage [4]. Yet, direct viral cytotoxicity is unlikely to be the primary cause of pathology: hantaviruses are not cytopathic *in vivo* [3], and hantavirus infection alone does not affect the permeability of endothelial cells *in vitro* [5]. A second factor might be the host immune response. CD8+ T cells can *in vitro* increase the permeability of human endothelial cell monolayers [6] and high levels of these cells are observed during acute HPS [7]. Furthermore, immunoblasts consisting largely of CD8+ T cells are detected both in the lungs of HPS patients [3], [8] and in the kidneys of NE patients [9]. Additionally, tissue damage could be caused by the overproduction of cytokines by infected monocyte/macrophages, especially TNF-α that is known to increase vascular permeability. Increased levels of the cytokines TNF-α, IL-6, and IL-10 have been reported in NE patients [10], [11] Furthermore, high levels of cytokine-producing cells are seen in the lungs of HPS patients [12], and the pulmonary fluid of HPS patients appears to be exudative in nature [13].

The role of different factors in hantavirus pathogenicity is best evaluated in a model system. In their natural hosts, hantaviruses cause an asymptomatic and persistent infection with no apparent pathology, and therefore, the use of rodent-based models is limited. Syrian hamsters infected with either Andes (ANDV) or Maporal virus do develop a disease similar to HPS, while another HPS-causing hantavirus, Sin Nombre, infects the hamsters asymptomatically [14]–[17]. HFRS hantaviruses are non-pathogenic to hamsters even at very high doses, and although suckling mice can be infected by HFRS hantaviruses, the disease does not resemble the disease in humans [18]. The first attempts to establish a monkey-based model were not successful either, probably due to the use of cell culture-adapted virus. The adaptation of wild-type PUUV (passaged in colonized bank voles) to cultured primate Vero E6 cells leads to genetic and phenotypic changes [19], [20]: the cell culture–adapted variant is noninfectious to bank voles. Many species of nonhuman primates develop an antibody response to PUUV or Prospect Hill virus (PHV) infection, but lack disease [21]. ANDV infection of macaques fails to cause in any disease, although there is an antibody response [22]. PUUV (cell culture –adapted) infection results in some mild symptoms, but without inflammatory reaction in the kidneys, which is in contrast to human NE-cases [23]. Cynomolgus macaques (*Macaca fascicularis*) infected with wild-type PUUV (strain Kazan-wt, passaged in colonized bank voles), however, develop a disease that mirrors closely NE in humans [24], including renal involvement and elevated cytokines. This model has been used successfully to study the protective potential of passive immunization against hantavirus infection [25]. Here, we present the histopathological examination of three monkeys described previously [24], focusing on characterization of the distribution of PUUV in tissues, and involvement of cytotoxic T-cells (CTLs).

## Results

All the three PUUV-infected monkeys lost their appetite during days 7 to 14 after the infection. During this period they were apathetic, and increased temperature was clearly demonstrated for one of the monkeys (#59) [24]. Similarly to human patients, the severity of the symptoms ranged from mild to severe. Kidney involvement was apparent since two of the monkeys developed proteinuria and hematuria. Nitric oxide responses were observed in all three monkeys, and elevated values of CRP and creatinine in two of the monkeys. Increased level of the cytokine IL-6 was seen in all three monkeys, and increased TNF-α in two monkeys [24].

**Figure 1:**
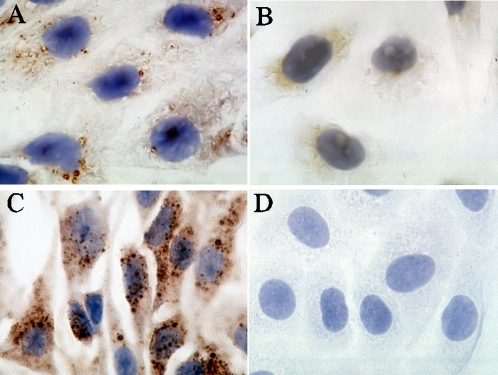
Specificity of the *in situ* hybridization probe. PUUV RNA was detected by *in situ* hybridization, and visualized with diaminobenzidine in PUUV-infected Vero E6 cells (A), while mock-infected cells (B) showed no signal. PUUV N antigen was detected by immunohistochemistry, visualized by diaminobenzidine, in PUUV-infected cells (C), while no staining was seen in mock-infected cells (D).

**Figure 2:**
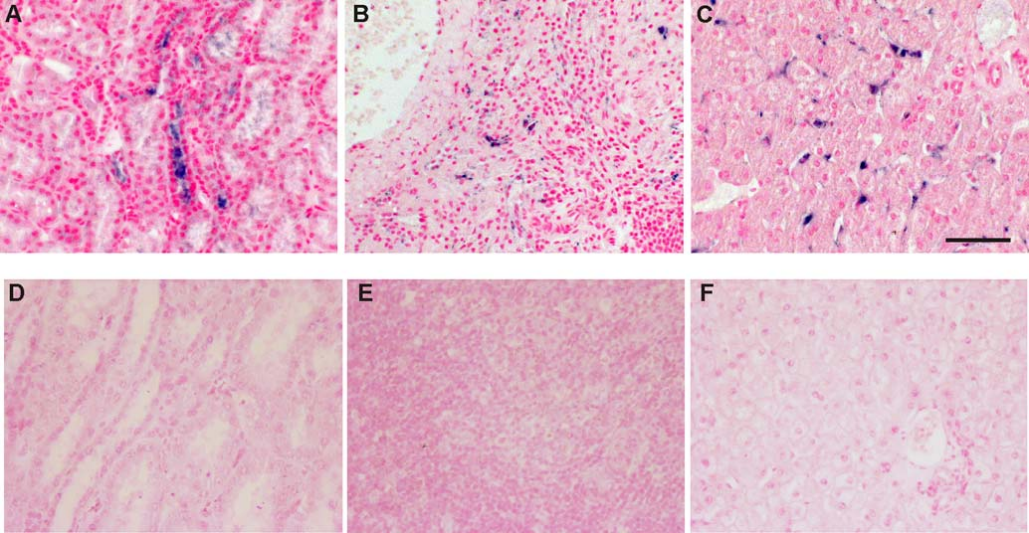
Detection of PUUV genome. PUUV S segment RNA was detected by *in situ* hybridization, and visualized with Ventana Blue in the kidney (A), spleen (B), and liver (C) of the monkey #59. In the spleen, PUUV RNA signal was mostly endothelial (capillaries) with some positive dendritic cells (B), and in the liver samples PUUV RNA was found in Kupfer cells (C). Scale bar corresponds to 50 µm. Negative control tissues were stained in parallel to the infected monkeys. No signal was detected in the kidney (D), spleen (E), or liver (F) of the negative control monkey.

In this study, we first analyzed the presence and location of PUUV genomes in tissue samples using *in situ* hybridization. The specificity of the probe was initially confirmed using Vero E6 cells: PUUV-infected cells as a positive control (Fig. 1A), and mock-infected cells as a negative control (Fig. 1B). No signal was observed in mock-infected cells. These cells were also stained with the polyclonal anti-PUUV-N antibody [26] to confirm the infection (Fig. 1C and 1D). PUUV RNA (negative strand) was detected in kidney (Fig. 2A), spleen (Fig. 2B) and liver (Fig. 2C) tissues of the infected macaques. These tissues were earlier found RT-PCR positive for PUUV RNA [24]. Tissue samples from a non-infected control monkey had no detectable signal (Fig. 2D–F). All monkeys had PUUV RNA in the kidneys, and the most severely affected monkey #59 gave the strongest signal (Fig. 2A, Table 1). Importantly, PUUV RNA was mostly seen in distal tubuli, and also in the lumen of the tubuli suggesting secretion of the virus into urine. In addition to kidney tubular epithelial cells, viral markers were occasionally found within capillary endothelium in kidney, liver and spleen. In the liver samples of two monkeys (#59 and #53) the virus was mostly found in Kupfer cells (Fig. 2C). In the spleen samples of these two monkeys, PUUV RNA was also detected, and the signal was mostly endothelial (capillaries) with some positive dendritic cells (Fig. 2B). The infected cells were identified based on the morphological characteristics of the cells. The heart and lung tissues remained negative in the *in situ* hybridization, although all the heart samples and one lung sample (from monkey #59) were RT-PCR positive [24]. In general, PUUV RNA was detected focally and at a low level in the tissues (except for the kidneys), and thus, the tissue stainings on a few sections might miss some of the positive foci.

**Table 1:** Detection of Puumala virus RNA and inflammatory cells in tissue samples

A: Puumala virus RNA (*in situ* hybridization*)			
	#25	#53	#59
KIDNEYS	++	++	+++
LIVER	−	+	++
SPLEEN	−	+	+(+)
LUNGS	−	−	−
HEART	−	−	−

B: Inflammatory cells** (in kidneys)				
	Neg	#25	#53	#59
CD20	+/−	+	++	+
CD45	+/−	+	++(+)	+(+)
CD3	+/−	+	+++	++
TIA-1	-	-	+++	++
the severity of infection increases from left to right				

* RT-PCR amplification of PUUV RNA was positive for all blood and tissue samples, except for the lung tissue samples of the monkeys #25 and #53

** Antigen scoring: − = no positive cells, + = rare positive cells, ++ = moderate number of positive cells, +++ = many positive cells

**Figure 3:**
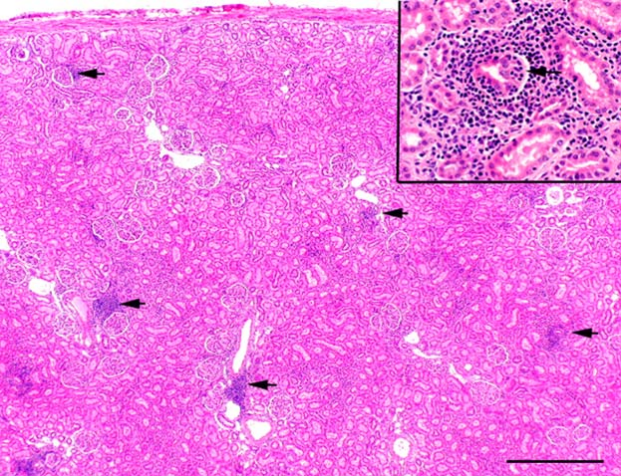
HE staining shows lymphoid cells (arrows) in the kidney tissue of the monkey #53. Scale bar corresponds to 500 µm. The magnification shows a damaged tubulus surrounded by inflammatory cells.

Histopathological examination revealed focal lymphocyte infiltrates mainly in the kidneys (Fig. 3), but some also in the lungs, and the heart. The tissue samples were collected 28 days after the infection, and it seemed that, at this time point, the viral replications had already ceased in the lungs and the heart, but the inflammation had not resolved fully yet. In the kidneys the inflammatory cell infiltrates were mostly tubulointerstitial, and while the infiltrates were absent in the least affected monkey #25, they were clearly detectable in monkeys #53 and #59. In the lungs interstitial pneumonia, thickening of alveolar septa and scattered lymphocytes were seen. Mild myocarditis was detected, while the liver and spleen appeared normal.

**Figure 4:**
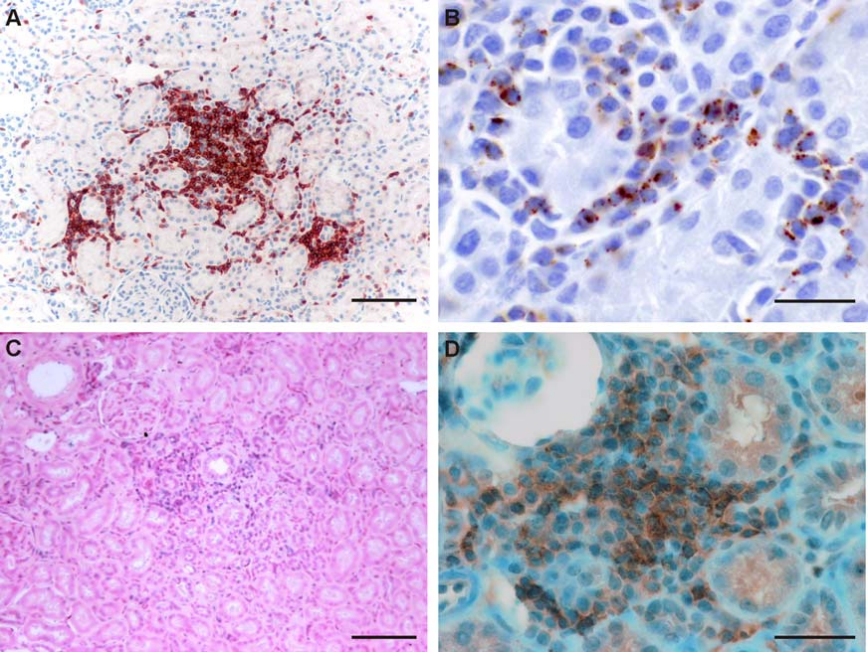
Characterization of the focal inflammatory cell infiltrates. These consist mainly of T-cells that are stained immunohistochemically with a polyclonal anti-CD3 antibody (A), and are visualized with Vector Red. A proportion of these T-cells is also positive for T-cell intracellular antigen-1 (TIA-1), a marker for active cytotoxic T cells (B). PUUV RNA, visualized with Ventana Blue (C), and PUUV N antigen, detected with a polyclonal anti-PUUV-N antibody, and visualized with diaminobenzidine (D), are found at the site of infiltrates in the kidneys of the monkey #53. Scale bars correspond to 100 µm (A and C) and 25 µm (B and D).

The number of the inflammatory cells was evaluated on an arbitrary scale. The inflammatory cells were predominantly CD3-positive T-cells, (Fig. 4A, Table 1), and a large proportion of these were of CD8-type (Fig. 4B). Staining was particularly pronounced at the sites of damaged tubuli (Figure 3, magnification). The highest amount of inflammatory cell infiltrates was detected in monkey #53. Notably, serially cut sections showed that PUUV RNA and N protein were detected at similar sites of tubular damage (Fig. 4C and D). Taken together, these results implied that the immunopathology caused by T-cells could be provoked directly by PUUV replication. Furthermore, the level of disease severity and the amount of virus detected in the infected macaques (Table 1) seemed to correlate. The least affected monkey (#25) was weakly positive for PUUV and only in the kidneys, where no inflammation above background was observed. Monkeys #53 and #59, which had clear clinical symptoms, also showed high level of inflammatory cell infiltrates and PUUV, especially in the kidneys. The difference in the distribution of viral markers and inflammatory cells in these two monkeys could be due to individual response and different kinetics of the infection.

## Discussion

In agreement with the current hypothesis on pathogenesis of hantavirus infection, both the virus and T-cells were detected in the two apparently ill macaques at the site of tissue damage. Autopsy data on human HPS cases caused by Sin Nombre virus infection have revealed that hantaviral antigens are mostly found in endothelial cells, but also other types of cells are positive, such as monocyte/macrophages [3]. PUUV antigen has been detected in the endothelium of the pituitary gland and in kidney tubuli of NE patients [27]. In one study on human kidney biopsies [28], PUUV antigen was detected in the cytoplasm of renal tubular epithelial cells with focal distribution in the cortical and medullary areas of the kidney. It therefore seems that in our study, PUUV RNA and antigen were detected in the monkey tissues in the same cell types and locations with focal distribution as in human autopsy tissue samples, confirming that Cynomolgus macaques infected with wt-PUUV mimic hantavirus disease in humans. Interestingly, the severity of human HFRS-cases ranges from mild (sometimes almost symptomless) to severe and to lethal, and similarly did the severity of the symptoms and pathological findings range also in the macaques. The important feature of this macaque model in comparison to other animal models for hantaviral diseases, is the finding of both the virus and the inflammatory cell infiltrates: the latter are absent in the naturally infected rodents hosts as well as in the earlier attempted macaque models [21]–[23]. Furthermore, the Syrian hamster model that works well with the HPS-causing ANDV [14] is apparently not applicable to HFRS-causing hantaviruses, which makes this macaque-model, at the moment, even more valuable.

Immunopathology is critical in hantaviral diseases. The HLA-B*3501 haplotype is associated with severe HPS caused by SNV infection implying involvement of CD8+ CTLs, and the number of SNV-specific CD8+ T cells correlates with disease severity [7]. Similarly, HLA-B8-DR3 is associated with severe outcome of PUUV infection [29], and the PUUV RNA level in such patients was particularly high, suggesting impaired handling of infection [30]. In general, this haplotype affects the early stages of immune activation by altering the balance of cytokines produced [31]. Furthermore, in kidney biopsies of NE patients the typical histopathological finding is acute tubulointerstitial nephritis, and inflammatory cell infiltrations [9]. These focal immunoblasts are similarly seen in the macaque model and they consist mainly of CTLs (Fig. 1D). In addition to T-cells, these infiltrations included some CD45- and CD20-positive B-cells (Table 1) in the monkey kidneys.

In NE patients, the CD8+ T cell response peaks at the onset of clinical disease, and decreases gradually within the next three weeks [32]. When the peak effector response subsides, the memory T cells start to emerge during the first weeks of convalescence. Thus, in our monkey tissue samples taken approximately 2–3 weeks after the onset of symptoms, the T cell response is already decreasing, and yet we find a high level of T-cell infiltrates, at least in the two more severely affected monkeys.

In general, the tissue samples from the PUUV-infected monkeys and from NE patients, have surprisingly low level of tissue damage despite clearly increased capillary leakage. It is known that hantavirus infection can increase the permeability of human endothelial cells *in vitro* by subtle mechanisms, and before apparent cytopathic effects [6]. Furthermore, hantavirus-specific CTLs can increase the endothelial cell permeability after antigen-recognition. It has been speculated that the mechanism of immunopathogenesis could be similar to the interactions between endothelial cells and CD8+ T-cells [33]. Normally, endothelial cells have higher-than-average tolerance for the cytolytic attack of CD8+ T-cells, and this down-regulation is mediated via binding of programmed death 1 –receptor (PD-1) on CTLs to the ligand PD-L1/L2 expressed on endothelial cells. An intriguing possibility is that in hantavirus infection this interaction could be disturbed either via excess amount of activated CTLs or alternatively, hantavirus infections could modulate the endothelial cell functions for example via changes in the expression of PD-L1/L2 [33]. Interestingly, renal tubular epithelial cells, another target of PUUV, can also down-regulate specific T-cell responses by increasing the expression of PD-L1 [34]. In the monkey tissues, hantavirus-infected endothelial and kidney epithelial cells were found at sites of T-cell infiltration supporting the hypothesis of virus-induced immunomodulation. This phenomenon, as well as other key issues of the pathology of hantavirus infection, may now be studied with the Cynomolgus macaque model.

## Materials and Methods

### Animals

The details of the experimental infection were described earlier [24]. The housing, maintenance and care of the animals was performed according to the relevant guidelines and requirements of the Swedish Institute for Infectious Disease Control. Briefly, the monkeys were inoculated intravenously with approximately 10^5^ bank vole 50% infective doses of PUUV strain Kazan-wt [19] in 1 ml of phosphate-buffered saline. Lung tissue material from infected bank voles was used as the inoculum. After 28 days of infection, the animals were sacrificed, and tissue samples collected.

### Immunohistochemistry

Samples of lung, heart, spleen, liver and kidney were dissected and stored frozen at −70°C. Tissues were fixed in 3% paraformaldehyde for 48 hours, dehydrated and paraffin-embedded. 4 µm sections were cut on slides. The sections were stained with hematoxylin-eosin for standard histopathological analysis. Immunohistochemical detection of CD20, CD45, CD3, and TIA-1 was done using VectaStain ABC kit with Vector Red as substrate. Detection of PUUV N protein was performed on the fully automated Ventana Discovery Slide stainer. Sections were first deparaffinized and pretreated in microwave oven in citrate buffer pH 6.0. Polyclonal antibody against Puumala virus N protein was used in 1∶100 dilution. Ventana iViewDAB kit was used for detection, and sections were counterstained with hematoxylin and postcounterstained with Bluing Reagent. Finally, the slides were rinsed and dehydrated before mounting with EuKitt mounting medium.

### *In situ* hybridization

A pcDNA3-plasmid containing PUUV S segment coding sequence (nt 61–1261) was linearised with BglII restriction enzyme. A probe hybridizing to nt 843–1261 of viral S segment RNA was in vitro transcribed and labeled with digoxigenin (DIG RNA labeling kit, Roche Applied Science). The specificity of the probe was first confirmed on VeroE6-cells that were infected with PUUV, strain Sotkamo, or mock-infected. In situ hybridization was carried out on the automated stainer. Sections were deparaffinized, fixed with RiboPrep and digested with Protease3. 250 ng of riboprobe was added to the slide and hybridized for 10 hours. Sections were washed, postfixed, endogenous biotin was blocked and hybridized probes were detected with BlueMap kit. Finally, slides were counterstained with nuclear fast red, rinsed, dehydrated and mounted permanently with EuKitt mounting medium. Tissue sections from a non-infected monkey were used as negative controls for both immunohistochemistry and *in situ* hybridization.

## References

[1] Nichol S, Beaty BJ, Elliott RM, Goldbach R, Plyusnin A, Fauquet CM, Mayo MA, Maniloff J, Desselberger U, Ball LA (2005). Bunyaviridae.. Virus taxonomy. VIIIth report of the International Committee on Taxonomy of Viruses.

[2] Vapalahti O, Mustonen J, Lundkvist Å, Henttonen H, Plyusnin A (2003). Hantavirus infections in Europe.. Lancet Infect Dis.

[3] Zaki SR, Greer PW, Coffield LM, Goldsmith CS, Nolte KB (1995). Hantavirus pulmonary syndrome. Pathogenesis of an emerging infectious disease.. Am J Pathol.

[4] Klingstrom J, Hardestam J, Stoltz M, Zuber B, Lundkvist Å (2006). Loss of cell membrane integrity in Puumala hantavirus-infected patients correlates with levels of epithelial cell apoptosis and perforin.. J Virol.

[5] Niikura M, Maeda A, Ikegami T, Saijo M, Kurane I (2004). Modification of endothelial cell functions by Hantaan virus infection: prolonged hyper-permeability induced by TNF-alpha of hantaan virus-infected endothelial cell monolayers.. Arch Virol.

[6] Hayasaka D, Maeda K, Ennis FA, Terajima M (2007). Increased permeability of human endothelial cell line EA.hy926 induced by hantavirus-specific cytotoxic T lymphocytes.. Virus Res.

[7] Kilpatrick ED, Terajima M, Koster FT, Catalina MD, Cruz J (2004). Role of specific CD8+ T cells in the severity of a fulminant zoonotic viral hemorrhagic fever, hantavirus pulmonary syndrome.. J Immunol.

[8] Nolte KB, Feddersen RM, Foucar K, Zaki SR, Koster FT (1995). Hantavirus pulmonary syndrome in the United States: a pathological description of a disease caused by a new agent.. Hum Pathol.

[9] Temonen M, Mustonen J, Helin H, Pasternack A, Vaheri A (1996). Cytokines, adhesion molecules, and cellular infiltration in nephropathia epidemica kidneys: an immunohistochemical study.. Clin Immunol Immunopathol.

[10] Linderholm M, Ahlm C, Settergren B, Waage A, Tarnvik A (1996). Elevated plasma levels of tumor necrosis factor (TNF)-alpha, soluble TNF receptors, interleukin (IL)-6, and IL-10 in patients with hemorrhagic fever with renal syndrome.. J Infect Dis.

[11] Mäkelä S, Mustonen J, Ala-Houhala I, Hurme M, Koivisto AM (2004). Urinary excretion of interleukin-6 correlates with proteinuria in acute Puumala hantavirus-induced nephritis.. Am J Kidney Dis.

[12] Mori M, Rothman AL, Kurane I, Montoya JM, Nolte KB (1999). High levels of cytokine-producing cells in the lung tissues of patients with fatal hantavirus pulmonary syndrome.. J Infect Dis.

[13] Bustamante EA, Levy H, Simpson SQ (1997). Pleural fluid characteristics in hantavirus pulmonary syndrome.. Chest.

[14] Hooper JW, Larsen T, Custer DM, Schmaljohn CS (2001). A lethal disease model for hantavirus pulmonary syndrome.. Virology.

[15] Milazzo ML, Eyzaguirre EJ, Molina CP, Fulhorst CF (2002). Maporal viral infection in the Syrian golden hamster: a model of hantavirus pulmonary syndrome.. J Infect Dis.

[16] Wahl-Jensen V, Chapman J, Asher L, Fisher R, Zimmerman M (2007). Temporal analysis of Andes virus and Sin Nombre virus infections of Syrian hamsters.. J Virol.

[17] Hooper JW, Ferro AM, Wahl-Jensen V (2008). Immune serum produced by DNA vaccination protects hamsters against lethal respiratory challenge with Andes virus.. J Virol.

[18] Kim GR, McKee KT (1985). Pathogenesis of Hantaan virus infection in suckling mice: clinical, virologic, and serologic observations.. Am J Trop Med Hyg.

[19] Lundkvist A, Cheng Y, Sjolander KB, Niklasson B, Vaheri A (1997). Cell culture adaptation of Puumala hantavirus changes the infectivity for its natural reservoir, Clethrionomys glareolus, and leads to accumulation of mutants with altered genomic RNA S segment.. J Virol.

[20] Nemirov K, Lundkvist A, Vaheri A, Plyusnin A (2003). Adaptation of Puumala hantavirus to cell culture is associated with point mutations in the coding region of the L segment and in the noncoding regions of the S segment.. J Virol.

[21] Yanagihara R, Amyx HL, Lee PW, Asher DM, Gibbs CJ, Jr (1988). Experimental hantavirus infection in nonhuman primates.. Arch Virol.

[22] McElroy AK, Bray M, Reed DS, Schmaljohn CS (2002). Andes virus infection of cynomolgus macaques.. J Infect Dis.

[23] Groen J, Gerding M, Koeman JP, Roholl PJ, van Amerongen G (1995). A macaque model for hantavirus infection.. J Infect Dis.

[24] Klingström J, Plyusnin A, Vaheri A, Lundkvist Å (2002). Wild-type Puumala hantavirus infection induces cytokines, C-reactive protein, creatinine, and nitric oxide in cynomolgus macaques.. J Virol.

[25] Klingström J, Stoltz M, Hardestam J, Ahlm C, Lundkvist Å (2008). Passive immunization protects cynomolgus macaques against Puumala hantavirus challenge.. Antivir Ther.

[26] Vapalahti O, Kallio-Kokko H, Närvänen A, Julkunen I, Lundkvist Å (1995). Human B-cell epitopes of Puumala virus nucleocapsid protein, the major antigen in early serological response.. J Med Virol.

[27] Hautala T, Sironen T, Vapalahti O, Pääkkö E, Särkioja T (2002). Hypophyseal hemorrhage and panhypopituitarism during Puumala virus infection: magnetic resonance imaging and detection of viral antigen in the hypophysis.. Clin Infect Dis.

[28] Groen J, Bruijn JA, Gerding MN, Jordans JG, Moll van Charante AW (1996). Hantavirus antigen detection in kidney biopsies from patients with nephropathia epidemica.. Clin Nephrol.

[29] Mustonen J, Partanen J, Kanerva M, Pietila K, Vapalahti O (1996). Genetic susceptibility to severe course of nephropathia epidemica caused by Puumala hantavirus.. Kidney Int.

[30] Plyusnin A, Horling J, Kanerva M, Mustonen J, Cheng Y (1997). Puumala hantavirus genome in patients with nephropathia epidemica: correlation of PCR positivity with HLA haplotype and link to viral sequences in local rodents.. J Clin Microbiol.

[31] Price P, Witt C, Allcock R, Sayer D, Garlepp M (1999). The genetic basis for the association of the 8.1 ancestral haplotype (A1, B8, DR3) with multiple immunopathological diseases.. Immunol Rev.

[32] Tuuminen T, Kekäläinen E, Mäkelä S, Ala-Houhala I, Ennis FA (2007). Human CD8+ T cell memory generation in Puumala hantavirus infection occurs after the acute phase and is associated with boosting of EBV-specific CD8+ memory T cells.. J Immunol.

[33] Terajima M, Hayasaka D, Maeda K, Ennis FA (2007). Immunopathogenesis of hantavirus pulmonary syndrome and hemorrhagic fever with renal syndrome: Do CD8+ T cells trigger capillary leakage in viral hemorrhagic fevers?. Immunol Lett.

[34] Waeckerle-Men Y, Starke A, Wuthrich RP (2007). PD-L1 partially protects renal tubular epithelial cells from the attack of CD8+ cytotoxic T cells.. Nephrol Dial Transplant.

